# Recombinant Expression and Bioprocess Optimization of *Priestia megaterium* α‐Amylase and Its Impact on Dough Fermentation Efficiency

**DOI:** 10.1002/cbdv.202500866

**Published:** 2025-07-12

**Authors:** Atacan Erdem, Kübra Akbulut, Mustafa Türker, Barış Binay

**Affiliations:** ^1^ Pak Gıda Üretim ve Pazarlama A.Ş. Kocaeli Türkiye; ^2^ Department of Bioengineering Gebze Technical University Gebze Kocaeli Türkiye; ^3^ BAUZYME Biotechnology Co. Gebze Technical University Technopark Gebze Kocaeli Türkiye

**Keywords:** α‐amylase, biomass, enzymes, fermentation kinetics, *Priestia megaterium*

## Abstract

α‐Amylase is a key hydrolytic enzyme in starch degradation, playing a crucial role in industrial bioprocesses such as dough fermentation. However, optimizing α‐amylase production remains challenging due to variations in microbial sources, culture conditions, and induction strategies. In this study, *Priestia megaterium* α‐amylase (*Pm*Amy) production was optimized for the first time based on the biomass/IPTG ratio in a controlled bioprocess. The impact of enzyme supplementation with equal quantity and enzymatic activity on dough fermentation was also evaluated to ensure consistent performance and effective application. Under the bioprocess conditions of 1.0 vvm airflow, 37°C, and 1000 rpm, a biomass‐to‐IPTG ratio was optimized as 20 g_biomass_ mmol_IPTG_^−1^ at pH 7.0. Fed‐batch fermentation was conducted at a specific growth rate of *µ* = 0.22 h^−1^ for 22 h, yielding an α‐amylase activity of 67.7 ± 4.0 U mL^−1^ at a cell concentration of 22.3 ± 5.3 g L^−1^ Dough fermentation trials demonstrated 99% efficiency compared to commercial α‐amylase, despite *Pm*Amy being in its primary recovery form. These findings highlight its potential for industrial baking applications. This study offers a scalable and sustainable enzyme production strategy, contributing to improved fermentation efficiency, product quality, and economic feasibility in food biotechnology.

## Graphical abstract

This study presents a scalable strategy for producing *Priestia megaterium* α‐amylase (*Pm*Amy) via fed‐batch fermentation, optimizing the biomass/IPTG ratio to enhance yield. With an activity of 67.7 U mL^−1^ at 22.3 g L^−1^ biomass, *Pm*Amy demonstrated 99% efficiency in dough fermentation, highlighting its industrial potential as a sustainable baking enzyme

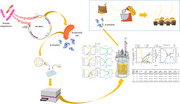

## 1 Introduction

Enzymes are highly sought after in industries due to their cost‐effectiveness and eco‐friendly advantages over chemical alternatives [1]. α‐Amylase, a glycosyl hydrolase and member of family 13 (EC 3.2.1.1), is an endo‐acting enzyme that catalyzes the cleavage of interior α‐1,4 glycosidic linkages in starch and other complex carbohydrates, releasing small subunits such as glucose, maltose, maltotriose, and low‐molecular‐weight dextrins [2]. One of the most important industrial enzymes, amylases contribute to around 25% of the market and are applied in many fields, including baking, food, detergent, biorefinery, textiles, paper, and medicine [3]. However, there is an ongoing interest in developing novel α‐amylases to meet the desired characteristics for specific applications from various microbes.

A wide range of organisms, including plants, animals, and microorganisms such as bacteria and fungi, can produce amylases. Among these, microbial amylases are particularly favored because of their rapid growth, ease of genetic manipulation, and high enzyme yields [4, 5]. Bacterial amylases are more widely used in industry, especially *Bacillus* strains such as *Bacillus amyloliquefaciens*, *Bacillus subtilis*, *Bacillus licheniformis*, and *Bacillus stearothermophilus* [6, 7]. However, their characteristic features such as pH and temperature optima, thermostability, metal ion dependency and expression yield vary even within the same genus and production process [8].

α‐Amylase production is typically achieved via solid‐state fermentation (SSF) and submerged fermentation (SmF) [9]. While SSF offers advantages in terms of reduced media cost, SmF is more commonly used because it provides superior control over key bioprocess variables such as pH, temperature, aeration, and agitation [10].

For downstream processing, proper selection of producers and optimization of bioprocess conditions are critical for cost‐effective production, especially on an industrial scale. *Escherichia coli* protein expression systems are the most popular for recombinant enzyme production. The ease of manipulation, availability of different strains and vectors, and the potential for rapid, cost‐effective, and high cell‐density fermentation are the key advantages of these systems [11, 12, 13, 14, 15].

T7 promoter‐based pET vectors, first reported for *E. coli* strain BL21 (DE3) by Studier, are the most commonly used systems for heterologous protein expression [16]. In these systems, the natural inducer lactose and the alternative chemical inducer isopropyl β‐d‐1‐thiogalactopyranoside (IPTG) are used to regulate the expression of recombinant genes and to develop bioprocesses. Gene expression is initiated upon the addition of lactose or IPTG, which induces the expression of T7 RNA polymerase, enabling the recognition of the T7 promoter. After full induction, almost all the cell resources are directed toward expressing the cloned gene. Hence, expression efficiency depends on adequate induction per cell. A cheap inducer, lactose, is often preferred over IPTG because of its natural occurrence [17, 18] and the known toxicity of IPTG to cells. However, recent advances in IPTG production processes have increased yields while reducing costs, making IPTG a more accessible option [19]. However, in both cases, inducer usage can pose bottlenecks, especially at the industrial scale. Inducer usage not only imposes an increase in the cost of production but also introduces practical challenges, such as monitoring cell density and determining the optimal induction point, which usually depends on host type. In addition, production yield can be different when the same inducer concentration is utilized for laboratory and industrial scale, and also, an increase in inducer concentration without an optimization study mediates significant toxicity to the cells [20]. Given these considerations, both lactose and IPTG were evaluated for induction in this study by systematically comparing different culture media before conducting bioprocess optimization and kinetic analyses.

α‐Amylases increase the availability of fermentable and reducing sugars in flour and dough, promoting yeast fermentation and formation of Maillard reaction products, which enhance bread flavor and improve crust color. In addition, amylases can decrease dough viscosity during gelatinization, thus extending the oven rise and contributing to an enhanced loaf volume. Finally, certain amylases exhibit anti‐staling properties by delaying crumb firming [21].

This study first conducted a high‐throughput screening of *Priestia megaterium* α‐amylase (*Pm*Amy) expression in *E. coli* BL21 (DE3) using different media, comparing lactose and IPTG as inducers. This was followed by bioprocess optimization, focusing on the biomass‐to‐inducer ratio to enhance expression of the *P. megaterium* amylase gene (*pmamy*) in high‐cell‐density cultures. Finally, the potential application of the produced enzyme in dough fermentation was investigated.

## 2 Experimental Section

### 2.1 Strains, Vectors, and Growth Cultures

The *pmamy* gene, carrying an N‐terminal 6 × His tag, was cloned into the pET‐28b(+) plasmid commercially, and the resulting plasmid was transformed into *E. coli* BL21 (DE3) cells by BAUZYME Biotechnology Company. The recombinant cells were kindly provided and used in this study. The control group of competent *E. coli* cells, which did not contain the plasmid, were also tested and showed no α‐amylase activity.

Luria‐Bertani (LB) medium was composed of 5.0 g L^−1^ yeast extract, 10.0 g L^−1^ peptone, and 10.0 g L^−1^ NaCl, while Terrific broth (TB) medium contained 23.6 g L^−1^ yeast extract, 11.8 g L^−1^ tryptone, 9.4 g L^−1^ K_2_HPO_4_, 2.2 g L^−1^ KH_2_PO_4_, and 4.0 g L^−1^ glycerol.

A glucose‐based medium was prepared containing 20.0 g L^−1^ glucose monohydrate, 2.5% (NH_4_)_2_PO_4_, 9.0 g L^−1^ KH_2_PO_4_, 6.0 g L^−1^ K_2_HPO_4_, and 0.3 g L^−1^ Na_3_‐citrate. A glycerol‐based medium was formulated to contain 20.0 g L^−1^ glycerol, 2.5% (NH_4_)_2_PO_4_, 9.0 g L^−1^ KH_2_PO_4_, 6.0 g L^−1^ K_2_HPO_4_, and 0.3 g L^−1^ Na_3_‐citrate. To support microbial growth and metabolic activity, these media were supplemented with 3.0 mL L^−1^ 1.0 M MgSO_4_·7H_2_O, 3.0 mL L^−1^ trace element (TE) solution, and 1.0 mL L^−1^ vitamin solution. The TE solution contained 0.5 g L^−1^ CaCl_2_·2H_2_O, 16.7 g L^−1^ FeCl_3_·6H_2_O, 0.18 g L^−1^ ZnSO_4_·7H_2_O, 0.16 g L^−1^ CuSO_4_·5H_2_O, 0.15 g L^−1^ MnSO_4_·4H_2_O, 0.18 g L^−1^ CoCl_2_·6H_2_O, and 20.0 g L^−1^ sodium ethylenediaminetetraacetic acid (Na‐EDTA). The vitamin solution consisted of 20.0 g L^−1^ thiamine hydrochloride and 1.0 g L^−1^ biotin.

The glucose feed solution used in fed‐batch fermentation contained 330.0 g L^−1^ glucose monohydrate, 49.5 mL L^−1^ 1.0 M MgSO_4_·7H_2_O, 49.5 mL L^−1^ TE solution, and 16.5 mL L^−1^ vitamin solution, which ensured sustained substrate availability and prolonged enzyme expression.

### 2.2 High‐Throughput Screening for Medium Selection

High‐throughput screening techniques were employed to determine the expression characteristics of α‐amylase in *E. coli* in the most popular media under two different inducers: lactose (Sigma‐Aldrich, Germany) and IPTG (Neofroxx, Germany). Screening was conducted using microplate readers (Varioskan LUX multimode Thermo Scientific, USA) within flat‐bottom, sterile 24‐ and 48‐well plates (Nest Biotechnology, China). For all media types, a 1.0% (v/v) inoculation was conducted in 1.0 mL total fermentation volume. Microplates were incubated at 37 °C and 600 rpm. Induction was performed at the end of the fourth h of incubation. After selecting the most suitable inducer, its concentration was optimized. Also, the effect of medium pH on cell growth and enzyme activity was analyzed by maintaining the pH level with citrate buffers (pH 4.0–5.0) and sodium phosphate buffers (pH 6.0–7.0).

### 2.3 *Pm*Amy Production in Bioreactors

The inoculum was prepared by incubating cells in 30 mL of LB medium for 24 h, and the culture was then inoculated into the bioreactor at a final concentration of 1% (v/v). The fermentation medium was glucose‐based and prepared with two different initial glucose concentrations: 20.0 and 40.0 g L^−1^. In fermentations with a total glucose concentration of 80 g L^−1^, 40 g L^−1^ of glucose was fed while maintaining a specific growth rate (*µ*) of 0.22 h^−1^ (Table 1).

**TABLE 1:** Glucose and IPTG concentrations in fermentation studies.

Ferm. methods	Initial glucose conc. (g L^−1^)	Fed glucose conc. (g L^−1^)	IPTG induction (mM)
**1**	20.0	—	0.5
**2**	40.0	—	0.5
**3**	40.0	40.0	0.5
**4**	40.0	40.0^a^	1.0

*Note*: 40 g L^−1^ of glucose was fed while maintaining a specific growth rate (*µ*) of 0.22 h^−1^. Fed glucose concentration: 330 g L^−1^. Each fermentation method was performed in replicates.

^a^ After 80 g L^−1^ glucose was consumed, automatic feeding was initiated based on 30% DO.

All fermentations were carried out in a 3‐L bioreactor (Biostat B, Sartorius AG, Germany) at 1.0 vvm airflow, 37°C, and 1000 rpm, with pH controlled using 21% NH_4_OH. In Fermentation Methods 1, 2, and 3, IPTG induction was performed at a concentration of 0.5 mM during the mid‐logarithmic phase (at 9–10 h of fermentation) when the biomass concentration reached 4.5 g L^−1^. In Fermentation Method 4, the IPTG concentration was calculated using Equation (6), and 1 mM IPTG induction was carried out in a single step after a total of 80 g L^−1^ glucose was consumed. In all experiments, exhaust gas composition (O_2_ and CO_2_) was monitored online using Biopat Xgas (Sartorius AG, Germany).

### 2.4 Fermentation Kinetics of Bioreactors

Fermentation kinetics were calculated using mass balance equations developed for substrate consumption and biomass growth (Equations (1), (2), (3)) [22]. The mass balance equations used for biomass and product formation in the fed‐batch process are given below:

(1) $$\frac{dVX}{dt}=\mu VX$$

(2) $$\frac{dVS}{dt}=F\left(t\right)S_{i}-\left(\frac{1}{Y_{X/S}^{0}}\frac{dVX}{dt}+mVX\right)$$

Integrating Equation (1) yields the following biomass equation for the variable‐volume fed‐batch process:

(3) $$X=\frac{V_{0}X_{0}}{V}\exp\left(\mu t\right)$$

The symbols μ, $X_{0}$ and X represent the specific growth rate (h^−1^), initial biomass concentration (g L^−1^) and cell concentration (g L^−1^) at any given time, respectively. The symbols V and $V_{0}$ represent the volume (L) at any time and initial volume (L), respectively. F represents the feed rate (L h^−1^), while $S_{i}$ and $Y_{X/S}^{0}$ represent the substrate concentration in the feed solution (g L^−1^) and the maintenance‐corrected yield coefficient, respectively. The symbol *m* represents the maintenance coefficient (g gX^−1^ h^−1^). By neglecting the maintenance coefficient for fast‐growing microorganisms, the following feed rate equation (Equation 4) is derived from Equations (1)–(3):

(4) $$F\left(t\right)=\frac{\mu V_{0}X_{0}}{S_{i}Y_{x/s}}\exp\left(\mu t\right)$$

### 2.5 Determination of Cell Growth

Cell growth was monitored by measuring absorbance at 600 nm (*A*_600_). After fermentation, cells were centrifuged (Centrifuge 5804 R, Rotor F‐34‐6‐38, Eppendorf AG, Germany) at 8000 rpm at 4°C for 5 min, and the supernatant was removed. The pellet was resuspended in 10 mL of deionized water (dH_2_O), and the dry weight of the washed biomass was determined using a moisture analyzer (MA37, Sartorius, Germany).

### 2.6 High‐Performance Liquid Chromatography Analysis of Carbon Sources and Organic Acids

The supernatant obtained after centrifugation was filtered through a 0.45 µm filter (RC, Sartorius, Germany) before high‐performance liquid chromatography (HPLC) analysis. Glucose and glycerol concentrations were measured using a 6 µm, 8 × 300 mm column (SUGAR SH1011, Shodex Co. Ltd., Tokyo, Japan) at 50°C with 0.005 M H_2_SO_4_ as the mobile phase at an isocratic flow rate of 0.8 mL min^−1^. Organic acid concentrations were analyzed using an 8 µm, 7.7 × 300 mm column (Agilent Hi Plex H, Agilent Technologies Inc., Santa Clara, CA, USA) at 55°C, with 0.005 M H_2_SO_4_ as the mobile phase, at an isocratic flow rate of 0.6 mL min^−1^.

### 2.7 Determination of Total Nitrogen Content

The nitrogen content of the biomass was determined using the Kjeldahl method, as described by the AOAC [23]. The biomass was digested with sulfuric acid, and total nitrogen was analyzed using the Kjelflex system (Kjelflex‐360 Digestion System, Buchi Labortechnik AG, Flawil, Switzerland). To calculate the protein content, the nitrogen value was multiplied by 6.25, and the percentage protein content was determined [24].

### 2.8 Cell Disruption

For small‐scale experiments conducted in microplates, 1 mL of cell culture was centrifuged at 10 000 rpm at 10°C for 5 min, and the supernatant was discarded. The pellet was resuspended in 750 µL of 1.0 M sodium phosphate buffer (pH 7.0), then transferred to bead‐beating microtubes (ZR BashingBead Lysis Tubes, 0.1 and 0.5 mm, Zymo Research, USA) and then cooled at −20°C for 5 min. After cooling, the sample was homogenized at 7000 × *g* for 1 min using a homogenizer (MagNA Lyser, Roche, Switzerland).

For the cells obtained in bioreactor experiments, cell lysis was achieved by subjecting the suspension to 30 cycles of sonication (15 s ON/15 s OFF) at 63% AMPL power (Ultrasonic homogenizer CY‐500, JP Selecta, Spain). After lysis, the suspension was centrifuged at 16 000 rpm at 10°C for 10 min using a micro‐centrifuge (Micro 200R; Hettich, Germany). The supernatant was then used for enzyme activity analysis.

### 2.9 Determination of *Pm*Amy Activity

The blue iodine–starch method developed by Fuwa was used to determine α‐amylase activity [25, 26]. The standard calibration curve for α‐amylase measurements was established using starch solutions ranging from 0.5 to 2.0 g L^−1^ (Potato starch 101252, Merck Millipore, Germany).

(5) $$\begin{array}{ccc} & & \mathrm{Enzyme}\;\mathrm{activity}\;(U\;mL^{-1})=\frac{(A_{580}\;\mathrm{control}-A_{580}\;\mathrm{sample})÷A_{580}/\mathrm{mg}\;\mathrm{star}\mathrm{ch}}{5\;\min(\mathrm{incubation}\;\mathrm{time})\times \;0.04\;\mathrm{mL}} \end{array}$$

The enzyme activity was calculated using Equation (5). *A*_580_ (control) represents the absorbance without enzyme addition, *A*_580_ (sample) refers to the absorbance of the enzyme‐digested starch, and *A*_580_ per mg starch corresponds to the absorbance of 1 mg of starch, as determined from the standard curve. The assay incubation time was 5 min, and the enzyme volume used was 0.04 mL.

### 2.10 *Pm*Amy Purification

*Pm*Amy was purified for use in dough fermentation. After cell lysis, the supernatant containing *Pm*Amy was filtered through a 0.45 µm RC filter (Sartorius, Germany) to remove any remaining cell debris. The supernatant was loaded into a pre‐equilibrated HisTrap Ni‐NTA column (Cytiva, USA) and elutions were collected using imidazole gradients (100–500 mM) [27]. After His‐tag purification, collected fractions were confirmed by sodium dodecyl sulfate–polyacrylamide gel electrophoresis (SDS‐PAGE) [28]. A PD‐10 column was then used to desalt the purified enzyme solution with 50 mM sodium phosphate at pH 7.0 to remove imidazole. In addition, pure *Pm*Amy was tested using a total viable count (TVC) assay to ensure the absence of microbial contamination [29].

### 2.11 Application in Dough Fermentation

In dough fermentation trials, *Pm*Amy was tested against commercial *Aspergillus oryzae* α‐amylase (Fungamyl 2500 Novozymes, Denmark). Since α‐amylase affects bread rheology and quality, its supplementation was standardized by testing in both equal enzyme amounts and equal enzymatic activity.

Special‐purpose flour from Bonatelli Food Company, Balıkesir (Türkiye) and special‐purpose wheat flour obtained from Ulusoy Flour Company, Samsun (Türkiye), were used. The dough was prepared using a 50:50 mixture of Tellioğlu and Ulusoy flour, with a total of 1.0 kg of flour per batch. The following ingredients were added based on the total flour weight: 1.5% salt, 3% fresh yeast, 59.5% water, and 0.2%–0.6% of the enzyme sample. The dough was left to ferment in a fermentation chamber at 32°C and 80% relative humidity (RH). After 1 h of fermentation, it was baked at 200°C for 30 min. Bread volume was measured using a TexVol BVM‐L450LC (Perten, Sweden). The falling number (FN) test was performed according to AACCI Approved Method 56‐81.03 and 22‐08.01 using an EKINOGRAPH FN 6 Viscoanalyzer (Ekinlab, Türkiye).

### 2.12 Statistical Analysis

Fermentations were conducted as biological duplicates with technical triplicates, and all enzymatic assays and bread‐making tests were repeated independently at least twice, each with technical replicates. Data presented in the graphs represent the mean values of all replicates. Standard deviations were calculated and are displayed as error bars. Fermentation kinetic parameters were derived from raw data. One‐way ANOVA was performed to assess homogenity of variances among experimental groups using Minitab (LLC 2021), with multiple comparisons conducted via the Tukey method at a 95% confidence level (*p* < 0.05). Different letters indicate statistically significant differences between groups.

## 3 Results and Discussion

### 3.1 High‐Throughput Screening for Medium Selection

**FIGURE 1:**
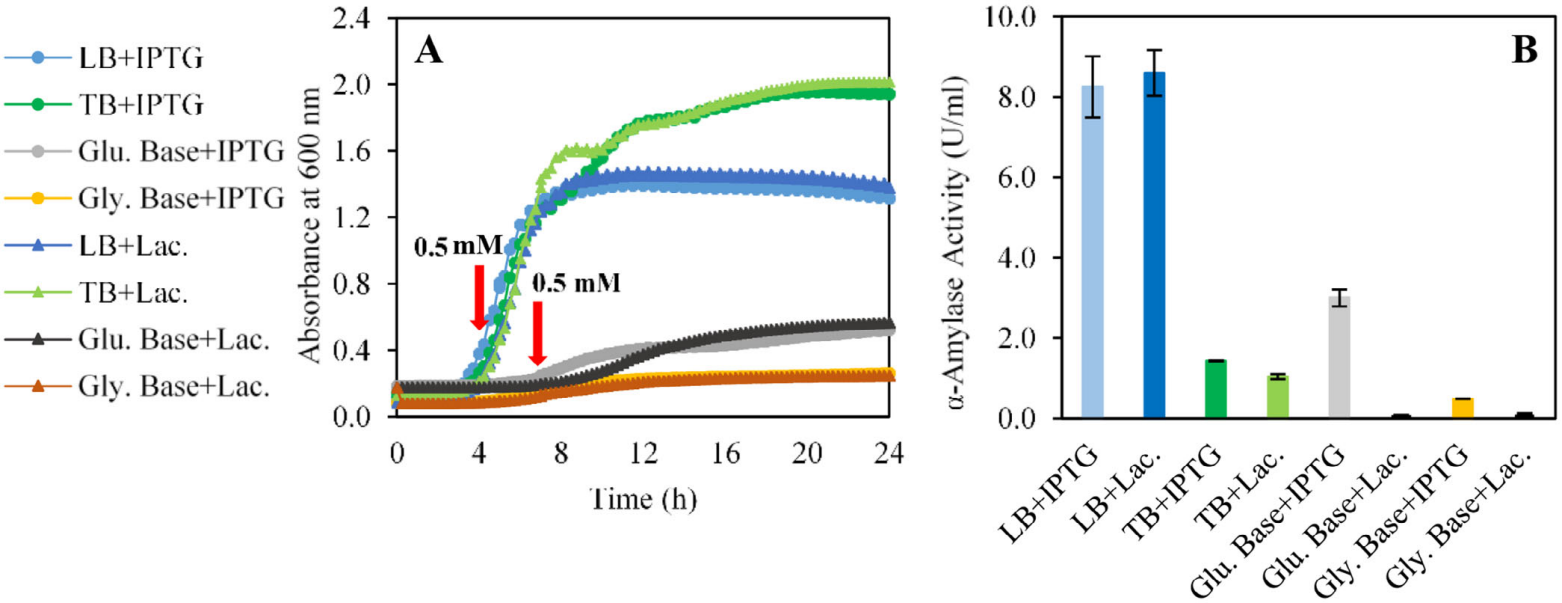
(A) Cell density profile of recombinant BL21 *Escherichia coli* strains grown in different media, measured over a 24‐h period. Induction (0.5 mM IPTG or lactose) was carried out at the fourth hour for LB and TB media, and at the seventh hour for glucose‐ and glycerol‐based media. (B) α‐Amylase activity levels measured under each condition. The symbol (+) denotes the type of inducer used.

Maximizing biomass production is a critical factor in enzyme synthesis, as it directly enhances the expression efficiency of individual cells in both extracellular and intracellular contexts [30]. One of the primary strategies to improve biomass yield is selecting the composition of the production media. In this study, we systematically evaluated commonly used media, including LB and TB, as well as defined media formulations such as glucose‐based and glycerol‐based media, by comparing lactose and IPTG as inducers in both complex and defined media to assess their efficacy in supporting biomass accumulation and enzyme synthesis (Figure 1).

As shown in Figure 1A, the highest biomass concentrations, 2.0 ± 0.0 and 1.9 ± 0.1, were achieved in TB medium under lactose and IPTG induction, respectively. LB medium supported a moderate biomass density of 1.3 ± 0.0, whereas defined media formulations exhibited limited biomass production likely due to acidic shifts caused by glucose metabolism. Specifically, the glucose‐based medium yielded biomass concentrations of approximately 0.5 ± 0.0, likely due to the accumulation of organic acids as metabolic by‐products. Acidic by‐product secretion is common in microorganisms. However, their accumulation inhibits cell growth by decreasing the growth rate even at concentrations as low as 0.5 g L^−1^ and reduces the synthesis of recombinant proteins in aerobic high‐cell‐density cultures [31, 32]. Therefore, keeping their concentration under the inhibitory threshold is important. Under excess glucose conditions, the carbon flux imbalance leads to acetate excretion, which not only lowers pH but also disrupts glycolysis and the TCA cycle, thereby limiting biomass production [33, 34]. Studies demonstrate that this acidification effect is exacerbated by carbon source‐dependent extracellular pH shifts, where glucose metabolism induces a rapid pH drop, impairing microbial growth if pH recovery does not occur [35]. In addition, fluorescence‐based pH monitoring in *E. coli* biofilms has revealed that localized acidification significantly alters microbial activity [36]. This phenomenon highlights the need for advanced pH stabilization in defined media to mitigate acidification during fermentation.

IPTG and lactose remain the most widely used inducers for protein production in recombinant *E. coli* expression systems [37, 38]. Regarding enzyme activity, the highest levels were observed in LB medium, with IPTG and lactose induction yielding 8.3 ± 0.8 and 8.6 ± 0.6 U mL^−1^, respectively (Figure 1B). In comparison, the glucose‐based medium with IPTG induction exhibited an enzyme activity of 3.0 ± 0.2 U mL^−1^, while no activity was detected under lactose induction in both glucose‐based and glycerol‐based media. The lack of activity can be attributed to the preferential metabolism of glucose and glycerol over lactose, as lactose utilization requires the depletion of these carbon sources [39]. To effectively use lactose as an inducer, alternative carbon sources such as glucose and glycerol must first be depleted in the medium [40, 41, 42]. Cardoso et al. reported that lactose‐induced cultures exhibited a delayed induction phase compared to IPTG‐induced cultures, likely due to the necessity of lac permease activation for lactose transport. While final protein yields were comparable, lactose‐induced cultures required extended cultivation times, which could impact overall process efficiency [43]. These findings underscore the importance of selecting an appropriate medium and inducer to optimize enzyme production, as well as the need to synchronize biomass accumulation with induction phases. Since enzyme activity was observed in all media types when IPTG was used as the inducer, the following optimization experiments were conducted using IPTG.

**FIGURE 2:**
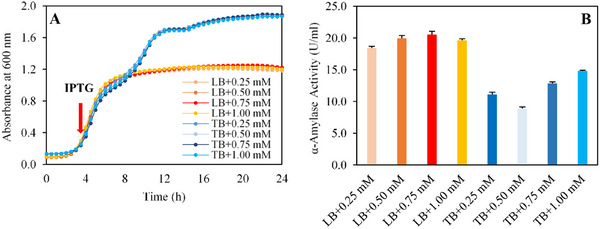
(A) Cell density profile of recombinant BL21 *Escherichia coli* strains in LB and TB media and different IPTG concentrations (0.25, 0.50, 0.75, and 1.00 mM) measured over 24 h. (B) α‐Amylase activity levels measured after induction with varying IPTG concentrations in LB and TB media.

Excessive IPTG concentrations in the medium can inhibit *E. coli* cells, while insufficient concentrations may fail to induce the desired enzyme expression [44]. To determine the optimal IPTG concentration for induction, a range of concentrations (0.25, 0.50, 0.75, and 1.00 mM) was evaluated in LB and TB media. The effects of IPTG concentration were assessed based on biomass production and enzyme expression (Figure 2).

As shown in Figure 2A, cells grown in TB medium reached an average cell density of 1.9 ± 0.0, while those in LB medium achieved a cell density of 1.2 ± 0.0. Notably, the tested IPTG concentrations were in a standard range and had no inhibitory effect on biomass in the tested media. The highest enzyme activity was obtained with LB media in all inducer concentrations, with a maximum activity of 20.0 U mL^−1^. On the other hand, in TB medium, increasing IPTG concentrations enhanced enzyme activity from 8.9 ± 0.3 to 14.8 ± 0.1 U mL^−1^ (Figure 2B). These findings indicate that the positive impact of IPTG concentration on enzyme expression becomes more pronounced at higher cell densities in TB medium. These results highlight the importance of optimizing IPTG concentrations to maximize enzyme yield, particularly in high‐cell‐density fermentations [45, 46, 47]. Therefore, the following optimization studies were carried out using LB medium due to its association with the highest observed enzyme activity.

**FIGURE 3:**
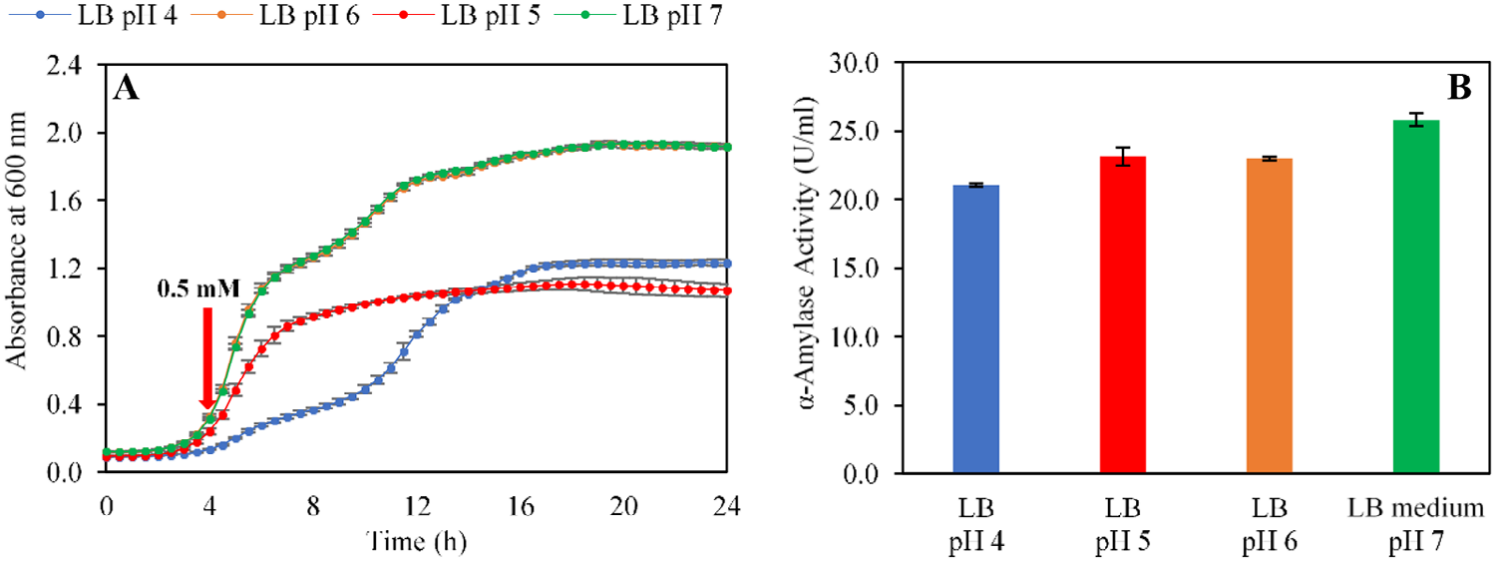
(A) Cell density profile of recombinant BL21 *Escherichia coli* strains grown at different pH values (4.0–7.0) in LB medium, monitored over 24 h. (B) α‐Amylase activity measured at different culture pH levels (pH 4.0, 5.0, 6.0, and 7.0).

The pH of the culture medium is a critical factor in optimizing α‐amylase production and biomass accumulation. Therefore, cell growth and enzyme expression were tested in a pH range between 4.0 and 7.0 in LB media and results are shown in Figure 3.

Specifically, cells cultivated in LB medium at pH 7.0 achieved a significantly higher cell density (1.9 ± 0.0 Abs_600nm_) compared to other pH levels (Figure 3A). These finding underscores that a neutral pH is optimal for *E. coli* growth, whereas acidic conditions substantially decrease both growth rates and final biomass yield. Such results are consistent with previous studies indicating a sharp decline in bacterial growth under acidic conditions, particularly when the pH drops below 4.0, a threshold known to exert bactericidal effects on *E. coli* [48].

Regarding enzymatic performance, the maximum α‐amylase activity was recorded at pH 7.0, reaching 25.8 ± 0.5 U mL^−1^ (Figure 3B). This enhanced activity can be attributed to the deprotonation of aspartic acid (Asp) and glutamic acid (Glu) residues within the active site of *Pm*Amy, which facilitates optimal glycosidic bond cleavage by matching the molecular conformation of the substrate [49].

### 3.2 Optimization of Carbon Source and IPTG‐to‐Biomass Ratio in Bioreactor Production

LB and TB media are advantageous for small‐scale studies for analyzing cell kinetics and behavior due to their nutrient‐rich compositions. However, their complex substrate profiles make them unsuitable for large‐scale bioprocess applications, particularly in scenarios requiring cost‐effective enzyme purification. This limitation has led to a preference for defined media such as glucose‐based systems [50]. As previously noted, carbon sources like glucose in defined media can induce the production of organic acids, primarily acetate during *E. coli* cultivation [33, 34, 35, 36]. For studies requiring high glucose concentrations, adopting a fed‐batch strategy significantly reduces organic acid accumulation, thereby enhancing biomass yield. In bioreactor experiments, a feeding profile was established using Equation (4), calibrated to a specific growth rate (*µ* = 0.22 h^−1^), to control total glucose addition exceeding 40 g L^−1^.

**FIGURE 4:**
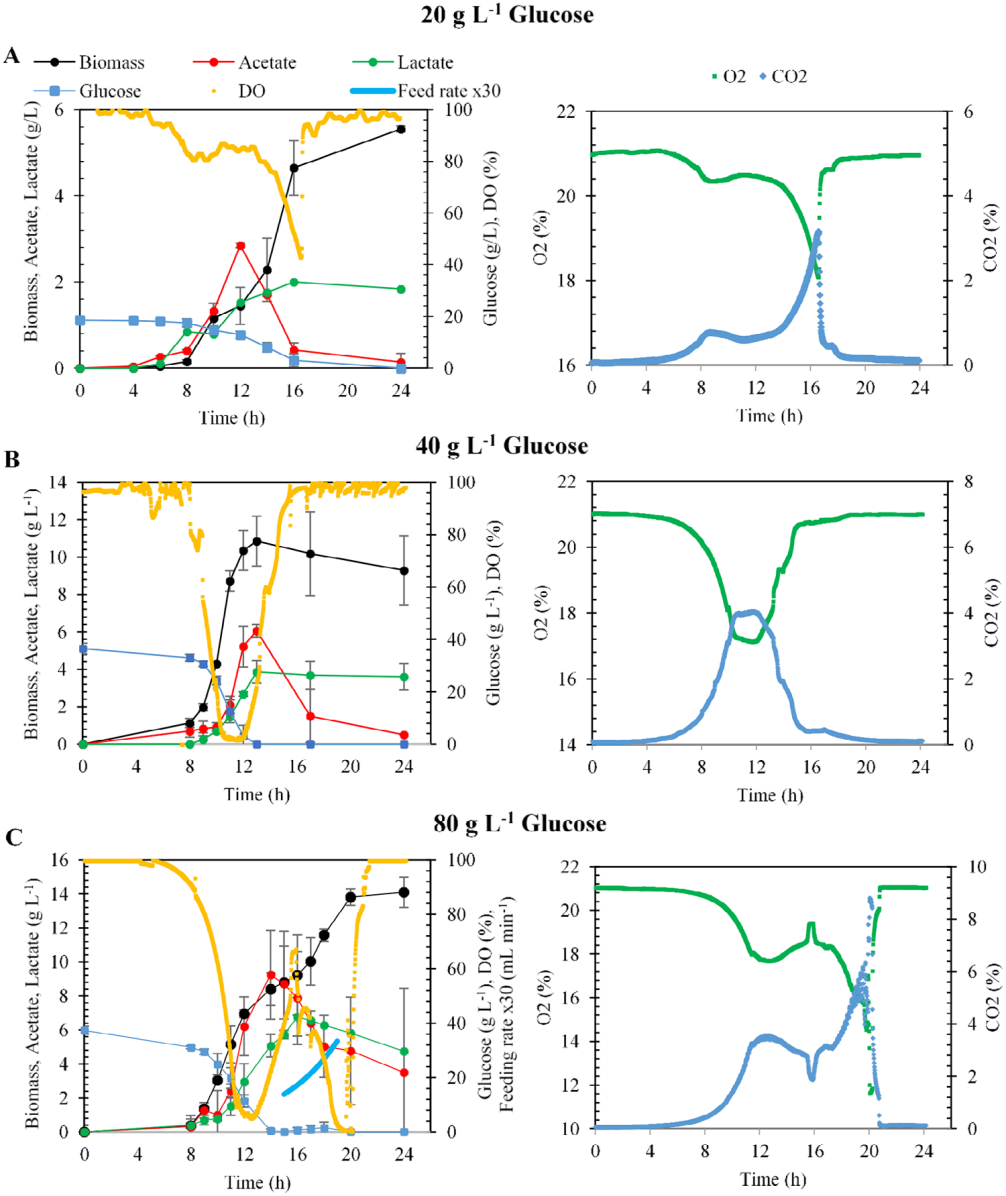
Fermentation trials conducted with recombinant *Escherichia coli* strain in a 3‐L fermenter with glucose‐based medium containing: (A) 20.0 g L^−1^, (B) 40.0 g L^−1^, (C) 80.0 g L^−1^ glucose. At the 10th hour of fermentation, induction was initiated with 0.5 mM IPTG.

Bioreactor trials using glucose‐based media, conducted with varying IPTG concentrations and glucose levels of 20, 40, and 80 g L^−1^, highlighted the critical role of IPTG in optimizing enzyme production. As illustrated in Figure 4, the results demonstrated that IPTG significantly enhances enzyme yield, particularly in high biomass conditions. These findings underscore the necessity of precise IPTG calibration in bioprocessing workflows to maximize productivity in glucose‐rich media.

As depicted in Figure 4, biomass concentration exhibited a marked increase with higher glucose supplementation. Fermentation initiated with 20 g L^−1^ glucose achieved a biomass concentration of approximately 1.0 g L^−1^ at the onset of the exponential growth phase and 5.6 ± 0.1 g L^−1^ at the end of fermentation. Following IPTG induction, a notable reduction in cellular growth rate was observed, persisting for approximately 6 h. Analysis of dissolved oxygen (DO) levels and exhaust gas composition (CO_2_ and O_2_) indicated glucose depletion in the fermentation medium shortly after the 16th hour, leading to the cessation of cellular activity (Figure 4A). Moreover, the accumulation of organic acids correlated positively with increased glucose concentrations. In fed‐batch fermentations with 40 g L^−1^ glucose, glucose supplementation effectively mitigated biomass yield loss by reducing by‐product formation (Figure 4C).

Fermentations with 20.0 g L^−1^ yielded a substrate‐to‐biomass yield (*Y*_X/S_) of 0.3 ± 0.0 g_biomass_ g_glucose_^−1^. The maximum specific growth rate (*µ*) was determined to be 0.6 ± 0.0 h^−1^, while α‐amylase activity at the end of fermentation was measured as 37.3 ± 2.2 U mL^−1^. Similarly, fermentations with 40.0 g L^−1^ glucose attained a maximum biomass concentration of 10.9 ± 1.3 g L^−1^, with a *Y*_X/S_ of 0.4 ± 0.0 g_biomass_ g_glucose_^−1^, and a specific growth rate of 0.71 ± 0.0 h^−1^. Under these conditions, α‐amylase activity reached 75.0 ± 4.0 U mL^−1^, representing the highest enzymatic activity observed. In fermentations conducted with 80.0 g L^−1^ glucose, biomass concentration peaked at 14.1 ± 0.9 g L^−1^ with a *Y*_X/S_ of 0.2 ± 0.0 g_biomass_ g_glucose_^−1^. The maximum specific growth rate was measured at 0.7 ± 0.0 h^−1^, while α‐amylase activity was 39.0 ± 2.0 U mL^−1^. At high sugar concentrations, the imbalance in carbon flux leads to overflow metabolism, resulting in the accumulation of by‐products such as acetate. This metabolic shift not only contributes to extracellular acidification but also disrupts key cellular processes by inhibiting glycolysis and the TCA cycle, thereby constraining enzymatic activity and overall metabolic efficiency [30]. To mitigate excessive acetate formation, which can negatively impact enzyme production, 40 g L^−1^ of the 80 g L^−1^ total glucose feed was supplied via fed‐batch feeding. This strategy effectively reduced acetate accumulation and its potential inhibitory effect on α‐amylase activity. This approach further supports the conclusion that IPTG insufficiency was a key limiting factor in the observed decrease in enzymatic activity.

**FIGURE 5:**
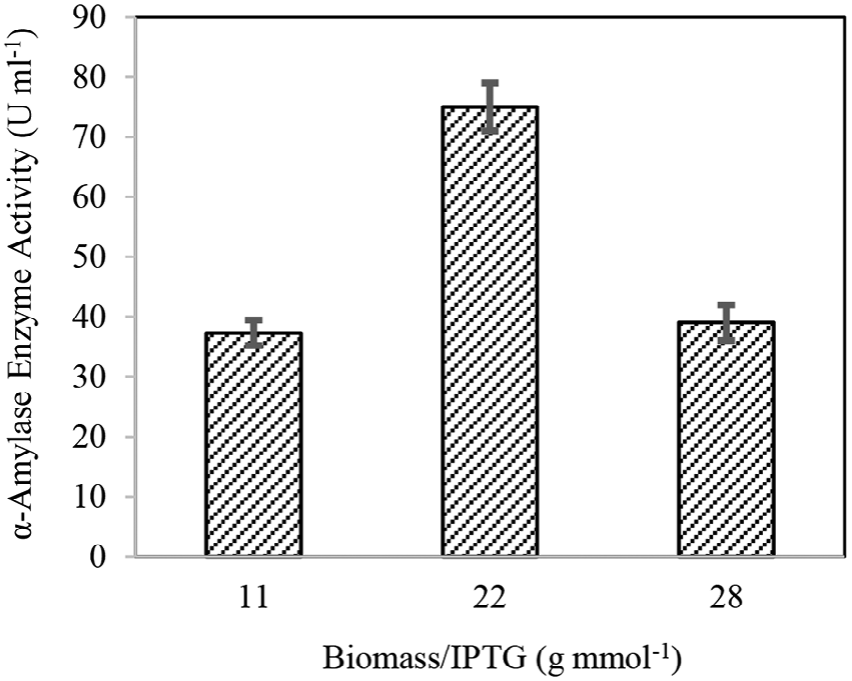
Correlation of α‐amylase activity with biomass (g L^−1^) to IPTG (0.5 mM) ratio obtained after conducting bioreactor production with glucose concentrations of 20, 40, and 80 g L^−1^.

These results demonstrate that α‐amylase activity increased alongside biomass concentration, reaching its peak at 10.9 ± 1.3 g L^−1^ under 40.0 g L^−1^ glucose conditions. Beyond this threshold, further biomass accumulation led to a decline in enzymatic activity. To better understand the relationship between biomass accumulation and IPTG availability, biomass concentration was normalized to IPTG feeding concentration (g mmol^−1^), allowing for a more detailed analysis, as illustrated in Figure 5.

Recent studies have shown that the biomass/IPTG ratio plays a critical role in optimizing enzyme production, particularly in large‐scale fermentation. An optimal ratio must be maintained to balance high biomass yields and effective induction, as excessive biomass can lead to a metabolic burden and reduced enzyme activity [51, 52]. In high‐biomass fermentation, IPTG has proven to be more effective than lactose and significantly enhances enzyme yield when an appropriate biomass/IPTG ratio is achieved [53]. Figure 5 demonstrates that applying a fixed IPTG induction of 0.5 mM to varying biomass concentrations resulted in insufficient IPTG availability, leading to a decline in enzyme activity as biomass concentrations increased. This behavior underscores the intricate relationship between biomass levels and IPTG uptake in recombinant protein production systems. Studies have shown that at high cell densities, IPTG transport dynamics shift significantly, with rapid depletion from the medium and active accumulation within cells. These dynamics can lead to suboptimal intracellular inducer levels if IPTG concentrations remain constant as biomass increases. Furthermore, the metabolic burden associated with elevated recombinant protein synthesis further highlights the need for precise inducer calibration to optimize protein expression while sustaining cellular activity [54, 55]. Biomass concentrations corresponding to glucose levels of 20, 40, and 80 g L^−1^ were 5.6 ± 0.1, 10.9 ± 1.3, and 14.0 ± 0.9 g L^−1^, respectively. When normalized to 0.5 mM IPTG these biomass concentrations corresponded to ratios of 11.1 ± 2.1, 21.7 ± 4.0, and 28.2 ± 3.0 g mmol^−1^, respectively.

Analysis of the normalized results revealed that maintaining a biomass‐to‐IPTG ratio of approximately 20 g mmol^−1^ was critical to preserving enzyme activity at elevated cell densities. Consequently, Equation (6) was derived to facilitate the application of this relationship in subsequent studies.

(6) $$20\;\left(g_{\mathrm{biomass}}\;\mathrm{mmo}{l_{\mathrm{IPTG}}}^{-1}\right)=\frac{\mathrm{Biomass}\;\mathrm{conc}.\;\left(g\;L^{-1}\right)}{\mathrm{IPTG}\;\mathrm{conc}.\;\left(\mathrm{mmol}\;L^{-1}\right)}$$

Similar studies have emphasized the critical importance of maintaining an optimal biomass/IPTG ratio. For example, during styrene monooxygenase (SMO) expression in *E. coli*, it was demonstrated that low cell concentrations could result in IPTG‐induced inhibition, indicating the necessity of calibrating inducer levels to avoid negative effects on cell viability [51]. Likewise, research has shown that high biomass concentrations can impose a significant metabolic burden on cells, adversely impacting recombinant protein yields. At elevated biomass levels, this burden has been linked to reduced enzyme activity, indicating that increasing biomass alone is insufficient for achieving optimal production [52].

**FIGURE 6:**
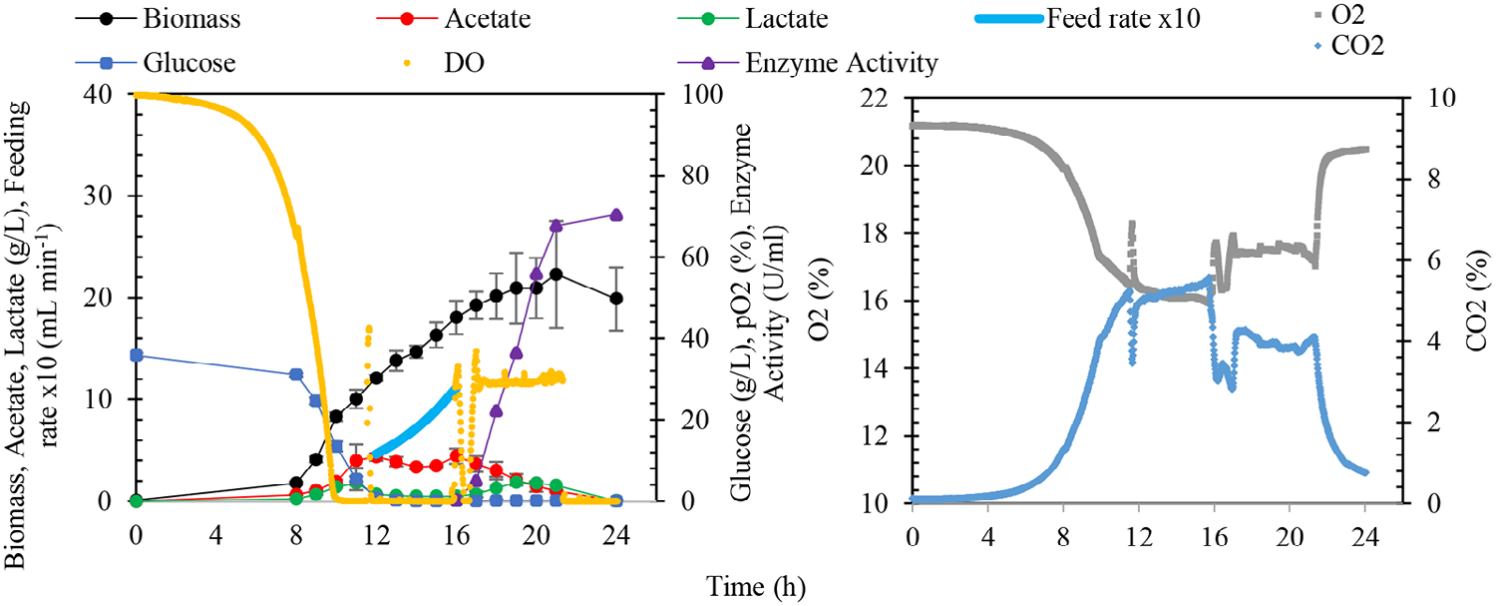
Fermentation trial of recombinant *Escherichia coli* strain conducted in a 3‐L fermenter with a glucose‐based medium containing 90 g L^−1^ glucose. The fermentation was initiated as a 40 g L^−1^ batch, followed by a fed‐batch phase at 40 g L^−1^ with a feeding *µ* = 0.22 h^−1^. At the 16th hour of fermentation after glucose depletion, IPTG induction (1 mM) was performed. Following induction, glucose feed solution was administered to automatically maintain the dissolved oxygen level at 30%.

To address these challenges, the fed‐batch operation was repeated using 80 g L^−1^ glucose, and the IPTG concentration was calculated using Equation (6). Approximately 1 mM IPTG was added at the 16th hour of fermentation (Figure 6). Enzyme production was monitored hourly throughout the induction phase to determine the point of maximum enzyme activity, ensuring alignment between IPTG levels and biomass concentration for optimal production efficiency.

The maximum cell concentration achieved during the fermentation was 22.3 ± 5.3 g L^−1^. Throughout the growth phase, the maximum specific growth rate was observed as 0.7 ± 0.0 h^−1^, with a substrate‐to‐biomass yield (*Y*_X/S_) of 0.3 ± 0.0 g_biomass_ g_glucose_^−1^. Following IPTG induction, enzyme activity increased steadily over 5 h, reaching a maximum of 67.7 ± 4.0 U mL^−1^. Beyond the fifth hour, no significant changes in enzyme activity were observed, as illustrated in Figure 6. This is the first study to optimize *Pm*Amy production using a biomass‐to‐IPTG ratio in a controlled fed‐batch fermentation process.

Table 2 provides a comparative overview of α‐amylase production in *E. coli* from various microorganisms reported in the literature, as well as the findings of this study, thereby highlighting its relative efficiency and performance under the described operational conditions.

**TABLE 2:** Fermentation conditions and final α‐amylase activities synthesized with *Escherichia coli*.

Source cells	Enzyme activity (U mL^−1^)	Fermentation mode	Volume	Growth medium		Reference
				pH	Temp. (°C)	
*Priestia megaterium*	67.7	Fed batch	3 L	7	37	This study
*Geobacillus thermodenitrificans* DSM‐645	75.7	N/A	N/A	6	37	[56]
*Pseudoalteromonas* sp. M175	141.1	Batch	Shake flask	N/A	37	[57]
*Paenibacillus* sp. SSG‐1	122.4	Batch	Shake flask	N/A	37	[38]
*Thermotoga petrophila*	12.8	Batch	Shake flask	7.5	37	[58]
*Pyrococcus woesei*	185	N/A	N/A	6	37	[59]
*Bacillus amyloliquefaciens* MTCC 610	29.5	Batch	3 L	7	37	[60]
*Bacillus subtilis* JN16	30	Batch	2 L	N/A	37	[61]

According to the previous characterization studies, *Pm*Amy shows the highest activity at a moderate temperature of 40°C and retains 55% of its initial activity when incubated at 40°C for 24 h. At the end of the process optimization, it reached a specific activity of 67.7 U mL^−1^ on potato starch in the current study. Characteristic properties of amylases, like optimum working pH (between pH 4 and 8) and temperature (between 30°C and 100°C), substrate specificity (dextran, starch, glycogen, maltotriose, and amylopectin) and kinetic properties, differ depending on the microbial source [62, 63]. Therefore, industries select the candidate amylase depending on these features according to their applications. For instance, the recombinant α‐amylase from *B. subtilis* JN16 exhibited an activity of 30 U mL^−1^ at its optimal temperature of 70°C and pH 5.5, showing its thermophilic nature. Although this enzyme presents activity at elevated temperatures, its activity remains significantly lower than *Pm*Amy under standard conditions. Similarly, a cold‐adapted α‐amylase from *Pseudoalteromonas* sp. M175 displayed a higher activity of 141.1 U mL^−1^ under optimized batch conditions, making it ideal for low‐temperature starch hydrolysis. However, such enzymes often exhibit compromised stability at higher temperatures, limiting their applicability in broader industrial processes [57]. Meanwhile, a thermostable α‐amylase from *Thermotoga petrophila* maintained functionality at extreme temperatures but exhibited substantially lower activity (12.8 U mL^−1^), demonstrating that thermostability alone does not necessarily correlate with higher catalytic activity [58].

These comparisons highlight the meaningful advantages of *Pm*Amy for industrial applications where moderate temperatures are required, such as in dough fermentation, due to its combination of high enzymatic efficiency and process simplicity. While cold‐adapted enzymes offer high activity under low temperatures and thermostable variants retain function under extreme conditions, *Pm*Amy strikes a favorable balance, achieving substantial activity under standard processing conditions, as shown in Table 2.

The production of α‐amylase from *P. megaterium*, as reported in this study, highlights the potential of this candidate as a novel enzyme for large‐scale production. Achieving an activity of 67.7 U mL^−1^ in fed‐batch fermentation demonstrates the efficacy of bioreactor optimization in improving enzyme yield. Notably, these activities were achieved without the addition of stabilizers or additives, further underscoring the inherent stability and efficiency of the expressed enzymes. Although IPTG has traditionally been considered a costly inducer for large‐scale applications, recent advancements in chemical synthesis and industrial bioproduction have significantly lowered its cost and improved availability [19, 39]. In this study, the potential limitation posed by IPTG was proactively addressed through biomass‐to‐inducer ratio optimization (20 g_biomass_ mmol_IPTG_^−1^), which enabled minimal IPTG usage while maintaining high expression levels. This approach not only reduces operational costs but also minimizes metabolic stress on host cells, increasing process robustness and reproducibility [43]. Therefore, with strategic optimization, IPTG remains a viable and effective inducer for industrial‐scale enzyme production. Ultimately, while bioprocess and host strain optimization remain critical factors, the use of IPTG—when integrated with targeted process design—offers a reliable platform for consistent and high‐yield enzyme expression suitable for industrial demands. In addition, potential risks associated with host‐derived proteins have been mitigated through advanced purification methods. However, the evaluation of bioprocess optimization for industrial production remains a key challenge. Therefore, the bioprocess data obtained in this study, particularly from bioreactor experiments, provide valuable insights into fermentation kinetics, as presented in Table 3.

**TABLE 3:** Fermentation kinetics and stoichiometric parameters for bioreactor experiments conducted with recombinant *Escherichia coli*.

Ferm. codes	IPTG (mM)	*µ*_max_ (h^−1^)	*Y*_X/S_ (g g^−1^)	*q*_AA,max_ (g gX^−1^ h^−1^)	*q*_LA,max_ (g gX^−1^ h^−1^)	*q*_S,max_ (g gX^−1^ h^−1^)	*r*_AA,max_ (g L^−1^ h^−1^)	*r*_LA,max_ (g L^−1^ h^−1^)	*r*_S,max_ (g L^−1^ h^−1^)	EA_max_ (U mL^−1^)
1	0.5	0.58^a^ ± 0.03	0.30^ab^ ± 0.01	0.60^a^ ± 0.06	0.30^a^ ± 0.07	1.34^a^ ± 0.22	0.76^a^ ± 0.08	0.38^d^ ± 0.03	2.53^b^ ± 0.27	37.3^b^ ± 2.2^b^
2	0.5	0.71^a^ ± 0.04	0.37^a^ ± 0.01	0.33^ab^ ± 0.03	0.14^a^ ± 0.01	2.02^a^ ± 0.09	3.12^a^ ± 0.44	1.22^b^ ± 0.04	9.39^a^ ± 0.21	75.0^a^ ± 4.0^a^
3	0.5	0.71^a^ ± 0.04	0.19^b^ ± 0.01	0.44^ab^ ± 0.03	0.27^a^ ± 0.05	2.21^a^ ± 0.24	2.21^a^ ± 0.09	1.74^a^ ± 0.07	8.47^a^ ± 1.03	39.0^b^ ± 2.0^b^
4	1.0	0.71^a^ ± 0.01	0.26^ab^ ± 0.04	0.24^b^ ± 0.08	0.12^a^ ± 0.01	2.19^a^ ± 0.28	2.38^a^ ± 0.67	0.72^c^ ± 0.02	10.05^a^ ± 0.78	67.7^a^ ± 4.0^a^

Abbreviations: *µ*_max_, maximum specific growth rate; EA_max_, maximum α amylase enzyme activity; *q*_AA,max_, maximum specific acetic acid production rate; *q*_LA,max_, maximum specific lactic acid production rate; *q*_S,max_, maximum specific substrate consumption rate; *r*_AA,max_, maximum rate of acetic acid production; *r*_LA,max_, maximum rate of lactic acid production; *r*_S,max_, maximum rate of substrate consumption; *Y*_X/S_, biomass yield.

One‐way ANOVA was performed to analyze equal variances with Minitab (LLC 2021), and grouping information using the Tukey method and 95% confidence level (*p* < 0.05). ^a^ and ^b^ indicate significant differences within the same column.

Analysis of the fermentation results revealed that the recombinant strain achieved a maximum specific growth rate of 0.71 h^−1^. The use of IPTG for induction in *E. coli* cultures influenced metabolic pathways and promoted the accumulation of organic acids, including lactic acid and acetic acid, which was likely caused by stress‐induced shifts in cellular metabolism [64]. However, optimizing the induction based on biomass/IPTG ratio significantly impacted these metabolic outcomes. Specifically, administering IPTG after biomass production reduced the accumulation of by‐products like lactic acid, as indicated by the observed reduction in the maximum lactic acid production rate [30]. Furthermore, inducing IPTG after complete glucose depletion improved the biomass yield (*Y*_X/S_) from 0.19 to 0.26 g g^−1^, highlighting the importance of precise control over induction timing in optimizing fermentation processes (Table 3).

Total biomass protein analysis at the end of the fermentation experiments revealed that protein content ranged between 80% and 85%, a remarkably high level compared to well‐known yeasts such as *Saccharomyces cerevisiae* and *Pichia pastoris*. These results align with previous studies which have demonstrated that bacteria, including *E. coli*, possess a high nitrogen content, making them well‐suited for recombinant protein production [65, 66, 67]. Nitrogen balance calculations further confirmed the efficiency of the process, with all balances exceeding 90%, as shown in Table 4. These findings underscore the advantages of bacterial systems in industrial bioprocesses while highlighting the critical role of metabolic and induction optimization in achieving superior yields and efficiency.

**TABLE 4:** Carbon, redox, and nitrogen balances of reactor experiments conducted with recombinant *Escherichia coli*.

Glucose (g L^−1^)	IPTG (mM)	Carbon balances (%)	Redox balances (%)	Nitrogen balances (%)
20.0	0.5	91.8^a^ ± 1.8	91.3^a^ ± 3.6	104.5^a^ ± 1.6
40.0	0.5	94.7^a^ ± 0.9	94.8^a^ ± 1.4	96.6^a^ ± 5.1
80.0	0.5	88.6^a^ ± 0.4	90.8^a^ ± 1.3	97.5^a^ ± 2.1
90.0	1.0	85.1^a^ ± 7.7	85.8^a^ ± 8.0	91.9^a^ ± 0.6

One‐way ANOVA was performed to analyze equal variances with Minitab (LLC 2021), and grouping information using the Tukey method (*p* < 0.05). ^a^ and ^b^ indicate significant differences within the same column.

These results confirm the consistency of high protein quantities. An examination of carbon and redox balances revealed an overall accuracy exceeding 90% (Table 4). However, deviations were noted, particularly in experiments utilizing the fed‐batch aimed at achieving high biomass production. This aligns with the findings of Demir et al. on *Ustilago maydis*, which showed that the elemental content of *U. maydis* changed during fermentation, especially in the fed‐batch process [68]. The changing elemental composition during fermentation may lead to imbalances in carbon and redox balances, resulting in deviations from the expected balance levels.

#### 3.2.1 Application in Dough Fermentation

**FIGURE 7:**
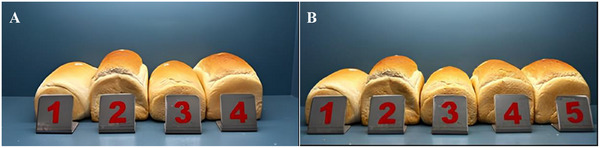
The breads made with *Pm*Amy and other α‐amylase enzymes. Part A represents equal amount of enzyme addition. A‐1 and A‐3: Control (without α‐amylase enzyme); A‐2: commercial α‐amylase (*Aspergillus oryzae*); A‐4: *Pm*Amy. Part B represents addition of an enzyme in equal activity. B‐1 and B‐3: Control (without α‐amylase enzyme); B‐2: commercial α‐amylase (*A. oryzae*); B‐4 and B‐5: *Pm*Amy.

The potential of rigorously purified *Pm*Amy in dough fermentation was analyzed by comparing it with a commercial enzyme widely used in the bakery industry. Before testing, the high purity of *Pm*Amy was confirmed via SDS‐PAGE, as shown in Supplementary Material Figure S.F.1. His‐tag purification was followed by PD10 column desalting to remove imidazole and maintain the enzyme in its desired buffer for stability. Therefore, after purification procedures, we expect minimal impurity and higher performance. Also, since the enzyme was intended for food applications, the enzyme sample was further tested using microbial growth assays to ensure the absence of microbial contamination (data not shown). Then, commercial α‐amylase from *A. oryzae* and *Pm*Amy were assessed in bread‐making trials using both equal enzyme quantities and matched activity levels (Figure 7; Table 5).

**TABLE 5:** Bread volume and falling number values obtained with control group, commercial α‐amylase, and *Pm*Amy.

Samples	Bread volume (mL)		Relative volume (%)		Falling number(s)	
	Equal amount	Equal activity	Equal amount	Equal activity	Equal amount	Equal activity
Control	1159^b^ ± 78	1159^b^ ± 80	77	88	393^a^ ± 21	393^a^ ± 21
Commercial α‐amylase (*Aspergillus oryzae*)	1515^a^ ± 29	1321^a^ ± 20	100	100	362^b^ ± 14	364^a^ ± 28
*Pm*Amy	1370^a^ ± 29	1309^a^ ± 36	90	99	394^a^ ± 27	392^a^ ± 28

One‐way ANOVA was performed to analyze equal variances with Minitab (LLC 2021), and grouping information using the Tukey Method (p < 0.05). ^a^ and ^b^ indicate significant differences within the same column.

Figure 7 shows that all breads treated with enzymes exhibited higher bread volumes compared to the control group. Among these, *Pm*Amy demonstrated a highly competitive performance in bread volume increase relative to a commercial enzyme, despite not being in a formulated form. When enzymes were applied at equal activity levels, *Pm*Amy achieved 99% efficiency in bread volume compared to the commercial α‐amylase, highlighting its comparable effectiveness. This highlights the potential for cost‐effective enzyme production and application in the baking industry. In the equal‐activity group, the commercial α‐amylase was used as a benchmark, yielding a bread volume of 1321 ± 20 mL, which was set as the reference value of 100% relative volume. *Pm*Amy closely matched this performance, achieving a bread volume of 1309 ± 26 mL, corresponding to a relative volume of 99.1% (Table 5). Although *Pm*Amy demonstrated a relative volume of 90% in the equal‐amount group, the observed difference compared to the commercial enzyme was not statistically significant, as detailed in Table 5. These findings emphasize the robustness and competitiveness of *Pm*Amy, indicating it as a potential alternative to commercial enzymes for enhancing bread quality in industrial baking applications.

As shown in Table 5, significant differences were observed in the FN values within the equal enzyme amount group, while no statistically significant differences were detected in the equal activity group compared to the control. In the equal enzyme amount group, the commercial amylase yielded an FN value of 362 ± 14 s, whereas *Pm*Amy demonstrated a slightly higher value of 394 ± 27 s. FN analysis measures changes in viscosity within a flour suspension, where higher enzyme activity leads to a more rapid decrease in viscosity. This suggests that the commercial amylase exhibits greater efficacy under equal enzyme amount conditions, as indicated by its lower FN value. It is important to note that the FN test is conducted at elevated temperatures, typically around 100°C, which places significant emphasis on the thermal stability of the enzyme. The reduced performance of *Pm*Amy under these high‐temperature conditions indicates lower thermal resistance compared to the commercial enzyme. However, as bread fermentation processes generally occur at more moderate temperatures (25°C–35°C), *Pm*Amy's higher activity under these conditions highlights its strong potential for applications in the baking industry [69].

Several studies have reported the use of α‐amylase in bread‐making to improve the rheological properties of dough. However, there are relatively few studies where valorized α‐amylases were produced using *E. coli* protein expression systems. In a study by Salem et al., the *B. subtilis* α‐amylase gene was expressed in *E. coli*, and bread‐making tests were conducted using two different enzyme concentrations [70]. The results showed that enzyme concentrations of 120 and 160 U kg^−1^ flour led to approximately 10 and 13% increases in bread volume, respectively. Another study demonstrated improvements in bread quality through the addition of α‐amylase derived from *Lactobacillus plantarum*, which exhibited an activity of 96.4 U mL^−1^ and enhanced bread volume by approximately 5% when applied at 6.7 U g^−1^ flour [71]. Similarly, a study by Ariaeenejad et al. investigated computationally derived α‐amylase from sheep rumen, which improved bread volume at an application rate of 0.008 U g^−1^ flour [72]. Furthermore, α‐amylase from the *B. subtilis* AS01a strain was compared to both a control (without enzyme) and commercial enzymes, resulting in approximately 55% and 17% greater increases in bread volume compared to the control and commercial enzymes, respectively [73]. As indicated, bacterial amylase sources were primarily preferred over yeast sources in bread making, demonstrating significant improvements in bread volume. In addition, supplemented amounts are very important for consistent activity and proper application of α‐amylase in dough fermentation. However, α‐amylases also have the capability to reduce hardness and enhance bread texture, shelf life, and chewiness. Therefore, further analysis is necessary to evaluate the effects of the candidate *Pm*Amy on bread properties during dough fermentation.

These innovations highlight the potential for *Pm*Amy as a promising, green effective alternative for industrial applications such as food biotechnology and dough fermentation, without requiring expensive formulation or purification processes.

## 4 Conclusion

This study successfully optimized the bioreactor‐based production of *Pm*Amy by evaluating batch and fed‐batch fermentation modes, particularly the influence of the biomass‐to‐inducer ratio. High‐throughput screening facilitated the selection of an optimal growth medium, while glucose‐based fed‐batch fermentation with IPTG induction at a biomass‐to‐inducer ratio of 20 g_biomass_ mmol_IPTG_^−1^ resulted in an α‐amylase activity of 67.7 ± 4.0 U mL^−1^ at a final biomass concentration of 22.3 ± 5.3 g L^−1^. This represents the first bioreactor optimization for *Pm*Amy production, providing a scalable and controlled approach to enhance yield and efficiency. The dough fermentation application of rigorously purified *Pm*Amy without a final formulation demonstrated a 99% efficiency in bread volume enhancement compared to commercial α‐amylase. Therefore, final formulations are suggested to enhance the stability and activity of *Pm*Amy. These findings emphasize the potential of *Pm*Amy as a cost‐effective alternative for the baking industry, particularly due to its high activity without additional formulation steps. Furthermore, this study provides valuable insights into recombinant enzyme production kinetics, emphasizing the importance of precise inducer calibration and controlled fermentation parameters for industrial scalability. To the best of our knowledge, this is the first report of *Pm*Amy production in bioprocesses, paving the way for its exploitation in industrial applications. Future research will aim to enhance *Pm*Amy's enzymatic properties through protein engineering, improve its stability under industrial conditions, and assess its compatibility with other amylases to broaden its applications. These efforts will contribute to the development of sustainable, high‐performance biocatalysts for food biotechnology and related industries.

## Author Contributions

**Atacan Erdem**: writing – review and editing, Writing – original draft, visualization, methodology, investigation, formal analysis, data curation, conceptualization. **Kübra Akbulut**: writing – review and editing, methodology, investigation, formal analysis, conceptualization. **Mustafa Türker**: writing – review and editing, conceptualization, supervision, project administration. **Barış Binay**: writing – review and editing, conceptualization, supervision, project administration.

## Conflicts of Interest

The authors declare no conflicts of interest.

## Supporting information




**Supporting File 1**: cbdv70233‐sup‐0001‐SuppMat.pdf

## Data Availability

Data are available from the corresponding author upon reasonable request.
