# Supplementary material for: Recombinant Expression and Bioprocess Optimization of Priestia megaterium α‐Amylase and Its Impact on Dough Fermentation Efficiency
Source: Chem Biodivers. 2025 Jul 12;22(11):e00866. doi: 10.1002/cbdv.202500866 (PMC12629164; doi:10.1002/cbdv.202500866)
Supplement: Supplementary file 1 — Supporting File 1: cbdv70233‐sup‐0001‐SuppMat.pdf [file CBDV-22-e00866-s001.pdf]

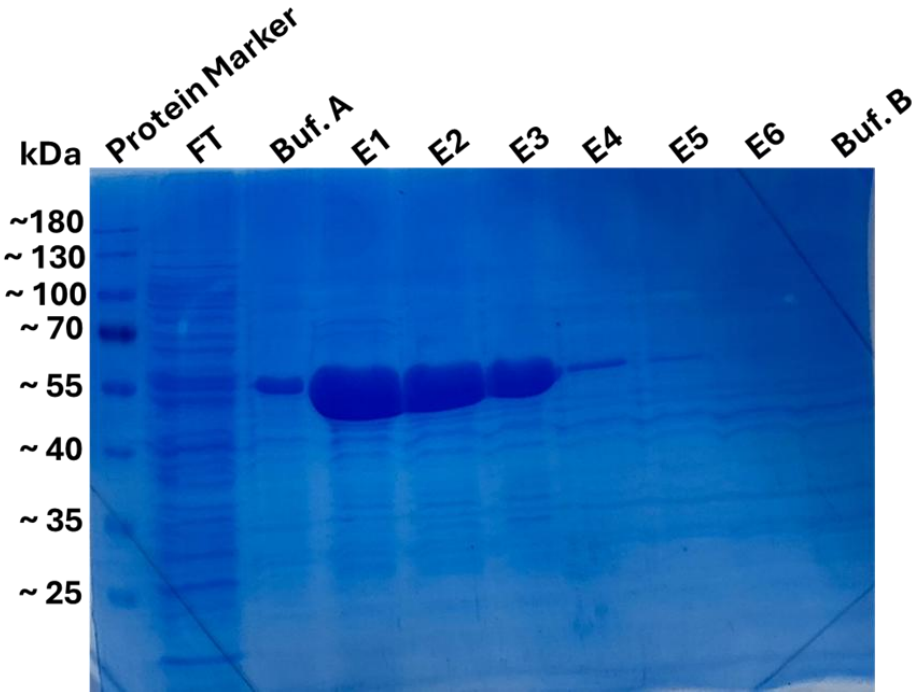

S.F.1. SDS-PAGE analysis of *Pm*Amy after HisTrap column purification FT: Flowthrough of column passing sample; Buf. A: sodium phosphate buffer having 30 mM imidazole; E1-E2: elutions having 100, 200, and 400 mM imidazole and Buf. B: sodium phosphate buffer having 500 mM imidazole.
